# OBESITY AND OUTCOME AMONG PATIENTS HOSPITALIZED FOR SARS-COV-2: A HOSPITAL-BASED STUDY IN INDONESIA

**DOI:** 10.21010/Ajidv17i2.5

**Published:** 2023-03-29

**Authors:** MASRUL Masrul, USMAN Elly, FEBRI Dewi Susanti

**Affiliations:** 1 Department of Nutrition, Faculty of Medicine, Universitas Andalas, Padang, Indonesia; 2 Department of Pharmacology, Faculty of Medicine, Universitas Andalas, Padang, Indonesia

**Keywords:** Obesity, Outcome, Hospitalized, SARS-CoV-2, Indonesia

## Abstract

**Background:**

Patients with obesity who have SARS-CoV-2 are at significant risk for developing serious clinical problems that need intensive care and have a bad prognosis.

**Aim:**

The aim of this study was to determine obesity and outcome among patients hospitalized for SARS-CoV-2 at the Indonesia’s national referral hospital.

**Methods:**

This study used a retrospective cohort. The study samples were SARS-CoV-2 patients who were treated by pulmonary specialists in the intensive room of Dr. M. Djamil Hospital Padang. The number of samples in this study was 106 subjects. Data analysis was performed using the Chi-square test, Kaplan-Meier, and Cox regression. P < 0.05 was significant, and the data were analyzed using the SPSS version 21.0 program.

**Results:**

The results of this study found obesity was associated with the outcome of SARS-CoV-2 patients treated at Indonesia’s national referral hospital (p<0.05, OR=3.55 (95% CI 1.44-8.71)). The mortality rate among patients hospitalized with SARS-CoV-2 at Indonesia’s national referral hospital with obesity was higher than non-obese; 82.7% and 57.4% respectively. The length of stay in patients with obesity was also shorter; 12 days compared to 19 days in non-obese.

**Conclusion:**

There was an association between obesity with mortality of SARS-CoV-2 patients in a national referral hospital in Indonesia. This study can provide input in the therapeutic management of patients with obesity so as to reduce the poor prognosis.

## Introduction

Infectious diseases and nutritional health are closely related under the SARS-CoV-2 pandemic conditions that are now in existence (Im *et al.*, 2020; Mehta, 2020). This is connected to the immune system, which is less than ideal if a person has a poor or abnormal nutritional status (Bold et al., 2020; Mentella *et al.*, 2021). In hospitalized SARS-CoV-2 patients, obesity is linked to prolonged hospitalization, complications, and even death (Abate *et al.*, 2021). Previous studies have found patients with obesity have a 56% - 67% chance of needing intensive care and hospitalization (Carrillo-Vega *et al.*, 2020; Hippisley-Cox *et al.*, 2020). Apart from that, the mortality rate ranges from 51% -75% during the treatment period (Carrillo-Vega *et al.*, 2020).

The condition of obesity is categorized as excess nutrition. In fact, obesity conditions also experience inflammation, which is a factor for exposure to SARS-CoV-2 v (Simonnet *et al.*, 2020). Obesity is related to metabolic syndrome system disorders such as diabetes mellitus, and other degenerative diseases that make the nutritional status of obesity to be classified as people who are vulnerable to the severity of SARS-CoV-2 (Cai *et al.*, 2020). Obese people have higher levels of pro-inflammatory adipokine leptin and lower levels of anti-inflammatory adipokine adiponectin (Lighter *et al.*, 2020). The immune system is modulated by this imbalance, which also causes difficulties in SARS-CoV-2 patients. As a result, the risk of death from SARS-CoV-2 patients rises (Chu *et al.*, 2020; Nindrea *et al.*, 2022).

The role of obesity in the death of hospitalized SARS-CoV-2 patients is still controversial. This can be seen from a previous study that stated that obesity did not increase the risk of death during hospitalization in patients with covid (Allard *et al.*, 2020). However, other studies found that obesity increases the risk of intensive care unit (ICU) admission and increased mortality in hospitalized SARS-CoV-2 patients (Chun *et al.*, 2020; Huang *et al.*, 2020).

Hospital nutrition issues are frequently overlooked, in part because weight, height, and dietary information are not recorded (Popkin *et al.*, 2020). The increase in nutritional requirements in conditions of sickness, trauma, stress, and other factors is frequently disregarded, while laboratory testing to measure nutritional status is frequently not carried out (Ruiz *et al.*, 2019). An early warning approach for identifying high-risk patients who need nutritional interventions to reduce the severity and fatality rates from SARS-CoV-2 is to assess the nutritional condition of SARS-CoV-2 patients who are hospitalized (Huang *et al.*, 2020; Popkin *et al.*, 2020). Based on the phenomena that have been described, the aim of this study was to determine obesity and outcome among patients hospitalized for SARS-CoV-2 at the Indonesia’s national referral hospital.

## Materials and Methods

### Study design and research sample

Retrospective cohort analysis was used in this study on SARS-COV-2 patients who were treated by pulmonary specialists in the ICU of Dr. M. Djamil Hospital Padang. Data were gathered from medical records between January and December 2021. A total of 100 subjects were represented as samples for this study. Convenience sampling is the technique that is used. Patients above the age of 18 years, those with SARS-COV-2 that were clinically severe and critical, and those who had weight and height information in the initial review of the medical record met the inclusion criteria for this study.

### Operational definition

Obesity was the study’s independent variable (obese, >25 kg/m^2^; non-obese, <25 kg/m^2^) (Lim *et al.*, 2017). The dependent variable was SARS-COV-2 patient mortality (death; survive) (Sjögren *et al.*, 2021).

### Research ethics approval

The Dr. M. Djamil Hospital Padang research ethics commission approved this study after conducting an ethical review (No. 28/ KEPK/ 2022).

### Data analysis

For categorical data, frequency and percentage were used, whereas the mean or median was used for numerical variables. The Chi-square test was used in the data analysis. In addition to calculating the median hospital stay for obese and non-obese patients, the Kaplan-Meier was utilized to observe the event (death). The equivalence of the survival curves was evaluated using the Log-rank test. In order to compare the length of hospital stays before events related to obesity, Cox regression and proportional hazards analysis were utilized. The data were analyzed using the SPSS version 21.0 program, and p-value< 0.05 was considered significant.

## Results

Subject characteristics (Table 1).

**Table 1:** Subject characteristics

Characteristics	Obese (n=52)	Non-obese (n=54)	p-value
**Age (years) , f(%)**			0.557^a^
<50	13 (59.1)	9 (40.9)	
50-59	13 (50.0)	13 (50.0)	
60-69	16 (50.0)	16 (50.0)	
≥ 70	10 (38.5)	16 (61.5)	
**Sex, f(%)**			0.053^a^
Male	25 (40.3)	37 (59.7)	
Female	27 (61.4)	17 (38.6)	
**Clinical severity, f(%)**			1.000^a^
Severe	8 (50.0)	8 (50.0)	
Critical	44 (48.9)	46 (51.1)	
**Inflammation marker, median (min-max)**			
D-Dimer (ng/mL)	3,494.50 (587-71,901)	3,297.00 (216-10,001)	0.709^b^
Procalcitonin (ng/mL)	0.74 (0.05-171)	0.27 (0.05-171.42)	0.092^b^
IL-6 (pg/mL)	64.35 (1.70-1,482)	42.10 (1.50-902.80)	0.406^b^
Ferritin (ng/mL)	1,107.58 (11.30-12,001)	1,201.00 (11.30-12,001.00)	0.309^b^
**Comorbid, f(%)**			
Hypertension	16 (47.1)	18 (52.9)	0.941^a^
Diabetes mellitus	22 (68.8)	10 (31.3)	0.014^a*^
Liver disease	1 (100.0)	0	n/a^a^
Cancer	1 (50.0)	1 (50.0)	1.000^a^
Pulmonary disease	1 (33.3)	2 (66.7)	1.000^a^
Kidney disease	9 (60.0)	6 (40.0)	0.525^a^
Immunodeficiency	0	1 (100.0)	n/a^a^
**Number of comorbid, f(%)**			0.259^a^
None	17 (40.5)	25 (59.5)	
1	22 (51.2)	21 (48.8)	
> 1	13 (61.9)	8 (38.1)	

* p<0.05 considered significant; a, Chi-square test; b, Mann-whitney test

Table 1 showed no differences in age, sex, clinical severity, D-Dimer, procalcitonin, IL-6, ferritin, comorbid hypertension, liver disease, cancer, pulmonary disease, kidney disease, and the number of comorbidities in the obese and non-obese group of patients SARS-CoV-2 (p>0.05). However, there were differences in comorbid diabetes mellitus in the obese and non-obese groups of SARS-CoV-2 patients (p<0.05).

Obesity and outcome among patients hospitalized SARS-CoV-2 (Table 2).

**Table 2:** Obesity and outcome among patients hospitalized for SARS-CoV-2 at Indonesia’s national referral hospital

Obese	Outcome		Total (f/%)	p-value	OR (95% CI)

	Death (f/%) (n=74)	Life (f/%) (n=32)			
Obese	43 (82.7)	9 (17.3)	52 (100.0)	0.009*^a^	3.55 (1.44-8.71)
Non-obese	31 (57.4)	23 (42.6)	54 (100.0)		Ref
**Total**	74 (69.8)	32 (30.2)	106 (100.0)		

* p<0.05 considered significant; a, Chi-square test; Ref, reference

Table 2 found that obesity was associated with the outcome of SARS-CoV-2 patients treated at Indonesia’s national referral hospital (p<0.05, OR=3.55 (95% CI 1.44-8.71)).

**Figure 1:**
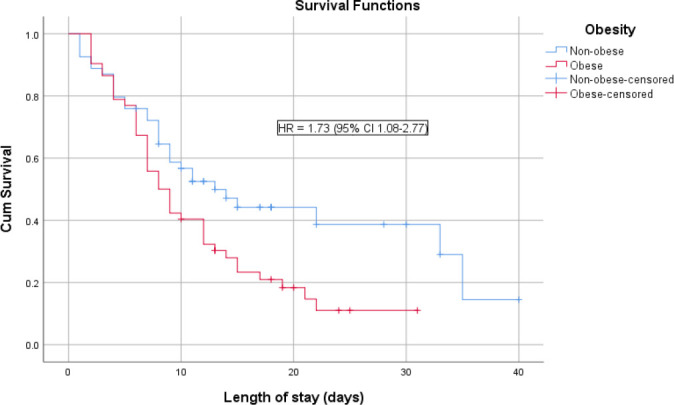
Survival curve for the outcome among patients hospitalized SARS-CoV-2 at Indonesia’s national referral hospital

Survival analysis for the outcome among patients hospitalized for SARS-CoV-2 at Indonesia’s national referral hospital (Table 3 and Figure 1).

**Table 3:** Survival analysis for the outcome among patients hospitalized for SARS-CoV-2 at Indonesia’s national referral hospital

Obese	Median length of stay (days) (min-max)	Death rate (%)	p-log rank test	HR (95% CI)
**Obese**	11.58 (9.13-14.04)	43 (82.7)	0.023*	1.73 (1.08-2.77)
**Non-obese**	19.19 (14.82-23.56)	31 (57.4)	Ref	Ref

* p<0.05 considered significant; Ref, reference

Table 3 shows the mortality rate among patients hospitalized with SARS-CoV-2 at Indonesia’s national referral hospital with obesity was higher than non-obese, namely 82.7% and 57.4%. The length of stay in patients with obesity was also shorter, namely 12 days compared to 19 days in non-obese. Obesity was associated with the outcome of SARS-CoV-2 patients treated at Indonesia’s national referral hospital (p<0.05, HR = 1.73 (95% CI 1.08-2.77)).

## Discussion

The results of this study found obesity was associated with the outcome of SARS-CoV-2 patients treated at Indonesia’s national referral hospital (OR=3.55). The mortality rate among patients hospitalized with SARS-CoV-2 at Indonesia’s national referral hospital with obesity was higher than non-obese, namely 82.7% and 57.4% respectively. The length of stay in patients with obesity was also shorter, namely 12 days compared to 19 days in non-obese.

In our study, it was established that both central and overall obesity are risk factors for SARS-CoV-2 infection-related hospitalization. Even with a small increase in weight, the established elevated risk is still present (Dreher *et al.*, 2020). Obese SARS-CoV-2 patients had a greater incidence of acute respiratory distress syndrome (ARDS) (46%) than non-obese patients (23%), according to a prior study (Rossi *et al.*, 2021). Another study found that SARS-CoV-2 patients with a BMI >40 kg/m^2^ needed intensive care 1.5 times more frequently than those with a BMI 25 kg/m^2^ (Djanas *et al.*, 2021).

High BMI was also regarded as a significant risk factor for the expense and length of hospital stays when it was discovered in a prior study of hospitalized patients that those with higher BMI were linked to increased morbidity and longer lengths of stay (Rossi *et al.*, 2021). Obese patients were found to spend more time in the ICU than patients of normal weight (Moriconi *et al.*, 2020).

Patients who are obese are more likely to get exacerbated from viral respiratory infections. According to a Swedish research of 1,649 SARS-CoV-2 patients receiving intensive care, a high BMI was linked to a higher chance of dying while receiving intensive care as well as a longer length of stay in the ICU (Peres *et al.*, 2020). BMI measurements are advised to be used in the severity evaluation for SARS-CoV-2 patients admitted to the ICU. From the result of the findings in this study, it has been shown that obesity can be an independent risk factor for severe outcomes in patients receiving intensive care. This is in agreement with the findings and submission of Nindrea (2023). The likelihood that obesity independently enhances the risk of severity of SARS-CoV-2 and other respiratory viral infections is raised by mounting evidence that obesity increases the risk of hospitalization, severity, and in some cases mortality from SARS-CoV-2 infection (Parikh *et al.*, 2020).

The strength of this study is that it was the first study conducted in one of Indonesia’s national referral hospitals about obesity and outcome among patients hospitalized with SARS-CoV-2. There are limitations on the current study. First of all, this study was retrospective and had a small sample size. The significance of obesity in the pathogenesis of SARS-CoV-2 infection also needs to be confirmed by more studies.

The implications of the results of this study were the establishment of an association between obesity, the length of stay, and mortality of SARS-CoV-2 patients in a national referral hospital in Indonesia. The therapeutic treatment of obese patients can benefit their prognosis with the help of this research.

## Conclusion

There was an association between obesity with mortality of SARS-CoV-2 patients in a national referral hospital Indonesia. This study can provide input in the therapeutic management of patients with obesity so as to reduce the poor prognosis.

## References

[1] Abate S. M, Chekole Y. A, Estifanos M. B, Abate K. H, Kabthymer R. H (2021). Prevalence and outcomes of malnutrition among hospitalized COVID-19 patients:A systematic review and meta-analysis. Clinical Nutrition ESPEN.

[2] Allard L, Ouedraogo E, Molleville J, Bihan H, Giroux-Leprieur B, Sutton A, Baudry C, Josse C, Didier M, Deutsch D, Bouchaud O, Cosson E (2020). Malnutrition:Percentage and Association with Prognosis in Patients Hospitalized for Coronavirus Disease 2019. Nutrients.

[3] Bold J, Harris M, Fellows L, Chouchane M (2020). Nutrition, the digestive system and immunity in COVID-19 infection. Gastroenterology and Hepatology from Bed to Bench.

[4] Cai Q, Chen F, Wang T, Luo F, Liu X, Wu Q, He Q, Wang Z, Liu Y, Liu L, Chen J, Xu L (2020). Obesity and COVID-19 Severity in a Designated Hospital in Shenzhen, China. Diabetes Care.

[5] Carrillo-Vega M. F, Salinas-Escudero G, García-Peña C, Gutiérrez-Robledo L. M, Parra-Rodríguez L (2020). Early estimation of the risk factors for hospitalization and mortality by COVID-19 in Mexico. PloS One.

[6] Chu Y, Yang J, Shi J, Zhang P, Wang X (2020). Obesity is associated with increased severity of disease in COVID-19 pneumonia:a systematic review and meta-analysis. European Journal of Medical Research.

[7] Djanas D, Yusirwan Y, Martini R. D, Rahmadian R, Putra H, Zanir A, Syahrial S, Nindrea R. D (2021). Survey data of COVID-19 vaccine side effects among hospital staff in a national referral hospital in Indonesia. Data in Brief.

[8] Dreher M, Kersten A, Bickenbach J, Balfanz P, Hartmann B, Cornelissen C, Daher A, Stöhr R, Kleines M, Lemmen S. W, Brokmann J. C, Müller T, Müller-Wieland D, Marx G, Marx N (2020). The Characteristics of 50 Hospitalized COVID-19 Patients With and Without ARDS. Deutsches Arzteblatt International.

[9] Hippisley-Cox J, Young D, Coupland C, Channon K. M, Tan P. S, Harrison D. A, Rowan K, Aveyard P, Pavord I. D, Watkinson P. J (2020). Risk of severe COVID-19 disease with ACE inhibitors and angiotensin receptor blockers:cohort study including 8.3 million people. Heart (British Cardiac Society).

[10] Huang Y, Lu Y, Huang Y. M, Wang M, Ling W, Sui Y, Zhao H. L (2020). Obesity in patients with COVID-19:a systematic review and meta-analysis. Metabolism:Clinical and Experimental.

[11] Im J. H, Je Y. S, Baek J, Chung M. H, Kwon H. Y, Lee J. S (2020). Nutritional status of patients with COVID-19. International journal of infectious diseases:IJID:Official Publication of the International Society for Infectious Diseases.

[12] Lighter J, Phillips M, Hochman S, Sterling S, Johnson D, Francois F, Stachel A (2020). Obesity in Patients Younger Than 60 Years Is a Risk Factor for COVID-19 Hospital Admission. Clinical Infectious Diseases:an Official Publication of the Infectious Diseases Society of America.

[13] Lim J. U, Lee J. H, Kim J. S, Hwang Y. I, Kim T. H, Lim S. Y, Yoo K. H, Jung K. S, Kim Y. K, Rhee C. K (2017). Comparison of World Health Organization and Asia-Pacific body mass index classifications in COPD patients. International Journal of Chronic Obstructive Pulmonary Disease.

[14] Mehta S (2020). Nutritional status and COVID-19:an opportunity for lasting change?. Clinical Medicine (London, England).

[15] Mentella M. C, Scaldaferri F, Gasbarrini A, Miggiano G. A. D (2021). The Role of Nutrition in the COVID-19 Pandemic. Nutrients.

[16] Moriconi D, Masi S, Rebelos E, Virdis A, Manca M. L, De Marco S, Taddei S, Nannipieri M (2020). Obesity prolongs the hospital stay in patients affected by COVID-19, and may impact on SARS-COV-2 shedding. Obesity Research &Clinical Practice.

[17] Nindrea R. D, Sari N. P (2022). How Does Family Planning Services Respond to the SARS-CoV-2 Pandemic in Indonesia?. Asia-Pacific Journal of Public Health.

[18] Nindrea RD. Impact of telehealth on the environment during the COVID-19 pandemic in Indonesia (2023). Asia Pacific Journal of Public Health.

[19] Parikh R, Garcia M. A, Rajendran I, Johnson S, Mesfin N, Weinberg J, Reardon C. C (2020). ICU outcomes in Covid-19 patients with obesity. Therapeutic Advances in Respiratory Disease.

[20] Peres K. C, Riera R, Martimbianco A. L. C, Ward L. S, Cunha L. L (2020). Body Mass Index and Prognosis of COVID-19 Infection. A Systematic Review. Frontiers in Endocrinology.

[21] Popkin B. M, Corvalan C, Grummer-Strawn L. M (2020). Dynamics of the double burden of malnutrition and the changing nutrition reality. Lancet (London, England).

[22] Rossi A. P, Gottin L, Donadello K, Schweiger V, Nocini R, Taiana M, Zamboni M, Polati E (2021). Obesity as a risk factor for unfavourable outcomes in critically ill patients affected by Covid 19. Nutrition, Metabolism, and Cardiovascular Diseases :NMCD.

[23] Ruiz A. J, Buitrago G, Rodríguez N, Gómez G, Sulo S, Gómez C, Partridge J, Misas J, Dennis R, Alba M. J, Chaves-Santiago W, Araque C (2019). Clinical and economic outcomes associated with malnutrition in hospitalized patients. Clinical Nutrition (Edinburgh, Scotland).

[24] Sjögren L, Stenberg E, Thuccani M, Martikainen J, Rylander C, Wallenius V, Olbers T, Kindblom J. M (2021). Impact of obesity on intensive care outcomes in patients with COVID-19 in Sweden-A cohort study. PloS one.

[25] Simonnet A, Chetboun M, Poissy J, Raverdy V, Noulette J, Duhamel A, Labreuche J, Mathieu D, Pattou F, Jourdain M &LICORN and the Lille COVID-19 and Obesity study group (2020). High Prevalence of Obesity in Severe Acute Respiratory Syndrome Coronavirus-2 (SARS-CoV-2) Requiring Invasive Mechanical Ventilation. Obesity (Silver Spring, Md.).

